# Navigated transcranial magnetic stimulation of the supplementary motor cortex disrupts fine motor skills in healthy adults

**DOI:** 10.1038/s41598-019-54302-y

**Published:** 2019-11-28

**Authors:** Severin Schramm, Lucia Albers, Sebastian Ille, Axel Schröder, Bernhard Meyer, Nico Sollmann, Sandro M. Krieg

**Affiliations:** 1 Department of Neurosurgery, Klinikum rechts der Isar, Technische Universität München, Ismaninger Str. 22, 81675 Munich, Germany; 2 TUM-Neuroimaging Center, Klinikum rechts der Isar, Technische Universität München, Munich, Germany; 3 Department of Diagnostic and Interventional Neuroradiology, Klinikum rechts der Isar, Technische Universität München, Ismaninger Str. 22, 81675 Munich, Germany

**Keywords:** Translational research, Premotor cortex

## Abstract

Navigated transcranial magnetic stimulation (nTMS) over the supplementary motor area (SMA) may impact fine motor skills. This study evaluates different nTMS parameters in their capacity to affect fine motor performance on the way to develop an SMA mapping protocol. Twenty healthy volunteers performed a variety of fine motor tests during baseline and nTMS to the SMA using 5 Hz, 10 Hz, and theta-burst stimulation (TBS). Effects on performance were measured by test completion times (TCTs), standard deviation of inter-tap interval (SDIT), and visible coordination problems (VCPs). The predominant stimulation effect was slowing of TCTs, i.e. a slowdown of test performances during stimulation. Furthermore, participants exhibited VCPs like accidental use of contralateral limbs or inability to coordinate movements. More instances of significant differences between baseline and stimulation occurred during stimulation of the right hemisphere compared to left-hemispheric stimulation. In conclusion, nTMS to the SMA could enable new approaches in neuroscience and enable structured mapping approaches. Specifically, this study supports interhemispheric differences in motor control as right-hemispheric stimulation resulted in clearer impairments. The application of our nTMS-based setup to assess the function of the SMA should be applied in patients with changed anatomo-functional representations as the next step, e.g. among patients with eloquent brain tumors.

## Introduction

The supplementary motor area (SMA) is a cortical region located in the premotor cortex, overlapping with Brodmann area 6. It can be divided into two subregions, the pre-SMA, located more anteriorly, as well as the SMA-proper, bordering on the primary motor cortex^1,2^.

Regarding functional aspects of the SMA, long lines of research have demonstrated its involvement in a variety of cognitive and motor-related processes. Multiple reviews exist in this regard^2,3^. Traditionally, its most noted role is the preparation and simulation of complex movement chains^2–4^. This is confirmed by lesion studies after ischemic events and by studies among patients who have undergone resections of brain lesions, which revealed a characteristic constellation of symptoms if the SMA is damaged: the so-called SMA syndrome usually presents as hemiparesis accompanied by varying degrees of mutism^5–7^. The SMA syndrome is usually considered to exist only temporarily and typically resolves over the course of weeks to months, which is likely associated with contralateral functional compensation^8–10^. The exact mechanism, however, remains largely unknown, and it is important to be aware of the fact that more detailed clinical examinations may be capable of detecting lasting deficits related to SMA damage, thus questioning the mere transient character of the SMA syndrome^11–14^. In addition, rare motor-related phenomena, such as the alien-limb syndrome, have also been reported in the past resulting from damage to the SMA. The alien-limb syndrome is characterized by a loss of conscious control of the afflicted limb, which may then move counter to the actual intent^12,15^. Furthermore, the role of the SMA in different cognitive processes such as mental object rotation, perception of effort, grip force scaling, and controlled coordination of movements has been explored repeatedly^16–19^. Other studies found evidence for projections interpreted to be associated with motor learning processes^20,21^.

Attempts at spatio-functional SMA delineation have been made using techniques such as functional magnetic resonance imaging (fMRI), magnetoencephalography (MEG), and positron emission tomography (PET)^22–25^. In one case study, MEG activity corresponding to voluntary movement preparation was recorded in a stroke patient possessing only one active SMA^23^. The study, however, mentions that MEG may at times be unable to record SMA activity due to both hemispheres canceling out each other’s recordable signal^23^. Whereas in two studies on 18 and 66 participants, fMRI has been used to some success in the localization of the SMA, there are reports of variable visibility in identification by resting-state fMRI^22,25^.

A modality to test or modulate SMA-related function is represented by transcranial magnetic stimulation (TMS). In therapeutic approaches a mild beneficial effect on the motor symptoms of Parkinson’s disease has been demonstrated (studies including 26 and 106 patients), seemingly arising from modulation of SMA excitability via repetitive stimulation, such as for example theta-burst stimulation (TBS)^26,27^. Other small-scale TMS studies (10 to 21 participants) exist on the study of functional connections between the SMA and (pre)motor areas^28–30^. A paired-pulse approach was used to create evidence for projections from the dorsal premotor area to the contralateral primary motor cortex^28^. Another study implies a difference in circuitry between premotor areas and the primary motor cortex^29^. Repetitive TMS has also been used over the SMA to heighten motor-evoked potentials^30^.

However, most of these studies used non-navigated TMS. Thus, correlations between measured effects and the exact spatial location of stimulation remained largely unclear. For multifarious TMS applications it has repeatedly been suggested that accurate neuronavigation of the stimulation, including optimal positioning and angulation of the stimulating coil with respect to cortical architecture, is important and may enhance precision and impact of stimulation^31,32^. Thus, particularly during preoperative application in modern neurosurgery, functional mapping by navigated TMS (nTMS) has emerged as a technology suited for mappings of sites including the motor cortex, language-related areas, or areas responsible for arithmetic processing^33–35^. Regarding further applications, the SMA has recently emerged as a potential new target structure for nTMS mappings; however, evidence is currently limited to one small series^36^. Yet, the need for mapping is clearly present in light of the questionable mere transient character of the SMA syndrome^11–14^. In the mentioned small series, a proof of concept was provided, showing that nTMS to the SMA can principally impact the performance of healthy adults in the Jebsen-Taylor Hand Function Test (JHFT) when delivering repetitive nTMS (rTMS) with 10 Hz^36^. However, whether other motor-related tasks or stimulation protocols are favorable for potential application of nTMS for mapping of the SMA has been beyond the scope of this previous investigation.

Against this background, the present study aims for systematic testing of nTMS effects on a variety of motor-related tasks by applying multiple stimulation protocols within healthy adults. In this framework, we decided to investigate the effects of stimulation with 5 Hz, 10 Hz, and TBS. Generally, repetitive stimulation is recommended over single-pulse TMS for the disruption of cortical processes in which precise timelines of activation are unknown^37^. 5-Hz stimulation has previously been shown effective in the mapping of various cortical functions. For example, multiple studies exist on its use in the mapping of cortical language function^38–41^. Furthermore, 10-Hz stimulation and TBS were chosen due to their different effects on cortical activity. 10-Hz stimulation is considered a paradigm leading to heightened neuronal activity^42^. On the other hand, TBS may have different neuromodulatory effects based on the exact mode of application, specifically with respect to the interval between bursts of stimulation. While continuous TBS is associated with dampening of cortical activity, intermittent TBS is believed to have facilitatory effects^43^. To identify possible stimulation-related effects, this study compares baseline task performance with performance under stimulation. The conclusions drawn from the present approach are supposed to aid in establishing an SMA mapping procedure analogous to previously used paradigms (e.g., mapping of language or calculation functions) and to better understand SMA functionality, its bilateral coordination, and its subcortical connectivity patterns.

## Results

### Cohort and mapping characteristics

**Figure 1:**
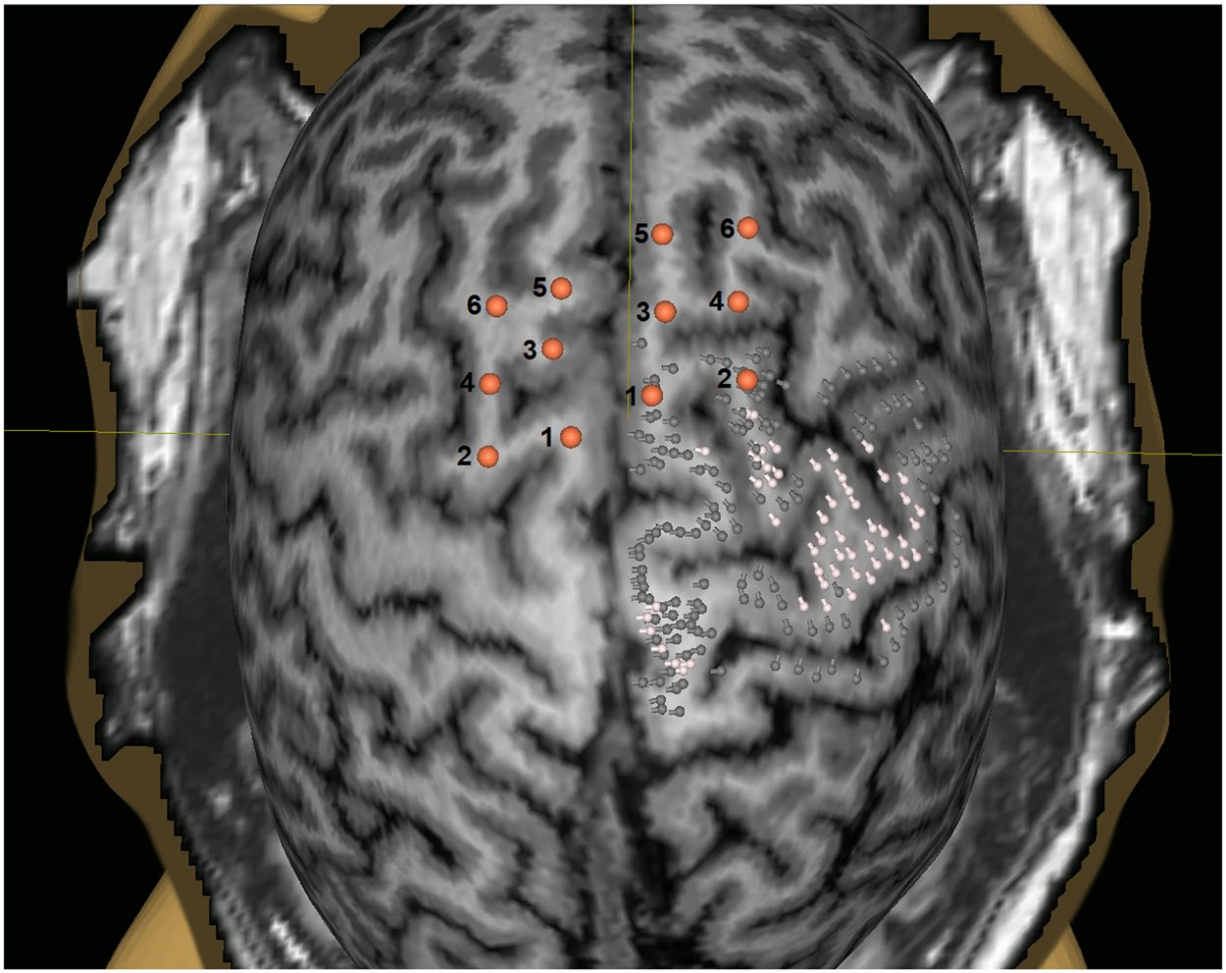
Showcase for stimulation target placement. This figure depicts the stimulation targets for one participant (six stimulation targets per hemisphere). On the right hemisphere, for additional information, the primary motor cortex as determined by motor mapping by navigated transcranial magnetic stimulation (nTMS) is shown in relation to the stimulation targets. Motor-positive points are displayed as white pins, motor-negative points as dark grey pins.

This study was performed in twenty healthy volunteers (8 males and 12 females, median age: 22.5 years, age range: 19–30 years), who were right-handed according to the Edinburgh Handedness Inventory (mean score: 74.5 ± 16.6 points). Motor and SMA mappings by nTMS were successfully performed in all participants during two separate appointments without technical problems or adverse events. Participants did not self-report any side effects of stimulation. According to randomization, the left hemisphere was stimulated during the first appointment in twelve participants. The average resting motor threshold (rMT) was 32.6 ± 5.5% (range: 23–42%) of the maximum stimulator output for the left hemisphere and 32.0 ± 4.2% (range: 25–42%) of the maximum stimulator output for the right hemisphere (p = 0.4967). Six stimulation targets per hemisphere were placed anteriorly of the primary motor cortex as determined by nTMS motor mapping (Fig. 1).

### Jebsen-Taylor Hand Function Test

#### Writing

Left hemisphere: Our analyses revealed significantly faster performances during stimulation in the majority of comparisons (p < 0.05; Table 1). Notably, the only comparisons not yielding significant dissociations were right-handed performances during stimulation with TBS (baseline test completion time [TCT] 10.8 ± 1.3 s; TBS TCT 10.5 ± 1.3 s [p = 0.1650]; Table 1). For right-handed performances during 10-Hz stimulation, stimulation to lateral targets led to slower TCTs than medial stimulation (medial group TCT 10.2 ± 1.3 s; lateral group TCT 10.5 ± 1.4 s [p = 0.0215]).

**Table 1:** Test completion times (TCTs) for the Jebsen-Taylor Hand Function Test (JHFT).

			Writing		Simulated page turning		Lifting small objects		Simulated feeding		Stacking checkers		Lifting light objects		Lifting heavy objects	
			TCT (s)	p-value to baseline	TCT (s)	p-value to baseline	TCT (s)	p-value to baseline	TCT (s)	p-value to baseline	TCT (s)	p-value to baseline	TCT (s)	p-value to baseline	TCT (s)	p-value to baseline
Left Hemisphere	Left hand	Baseline	22.7 ± 5.4		4.0 ± 0.8		5.6 ± 0.8		6.8 ± 1.1		3.3 ± 0.6		3.0 ± 0.4		2.9 ± 0.4	
		5 Hz	20.3 ± 4.1	0.0012	4.1 ± 0.5	0.2611	5.7 ± 0.6	0.8124	7.1 ± 1.0	0.3118	3.1 ± 0.5	0.0637	2.8 ± 0.3	0.0240	2.8 ± 0.3	0.0897
		10 Hz	19.9 ± 3.1	0.0001	4.1 ± 0.6	0.3683	5.8 ± 0.7	0.2943	7.0 ± 0.8	0.4749	3.3 ± 0.6	0.6742	2.8 ± 0.3	0.1650	2.8 ± 0.3	0.2774
		TBS	20.7 ± 4.3	0.0037	4.0 ± 0.6	0.7841	5.9 ± 0.7	0.1536	6.9 ± 0.8	0.5459	3.3 ± 0.5	0.8695	2.9 ± 0.4	0.3300	2.8 ± 0.4	0.6742
	Right hand	Baseline	10.8 ± 1.3		3.8 ± 0.7		5.2 ± 0.6		6.0 ± 0.8		3.2 ± 0.7		2.9 ± 0.4		2.8 ± 0.4	
		5 Hz	10.4 ± 1.3	0.0240	4.0 ± 0.7	0.2162	5.5 ± 0.7	0.1140	6.3 ± 0.6	0.1769	3.0 ± 0.4	0.5706	2.7 ± 0.3	0.1327	2.7 ± 0.3	0.5217
		10 Hz	10.4 ± 1.3	0.0441	4.0 ± 0.9	0.3683	5.7 ± 0.9	0.0266	6.4 ± 0.8	0.1429	3.0 ± 0.6	0.5459	2.7 ± 0.3	0.4091	2.7 ± 0.3	0.1650
		TBS	10.5 ± 1.3	0.1650	4.1 ± 0.7	0.0362	5.7 ± 0.9	0.0083	6.3 ± 0.5	0.1429	3.1 ± 0.5	0.6477	2.8 ± 0.4	0.9854	2.7 ± 0.3	0.9854
	Pooled hands	Baseline	16.8 ± 7.2		3.9 ± 0.7		5.4 ± 0.7		6.4 ± 1.0		3.2 ± 0.7		2.9 ± 0.4		2.8 ± 0.4	
		5 Hz	15.3 ± 5.9	<0.0001	4.0 ± 0.6	0.0747	5.6 ± 0.7	0.1922	6.7 ± 0.9	0.0892	3.1 ± 0.5	0.0817	2.8 ± 0.3	0.0056	2.7 ± 0.3	0.0842
		10 Hz	15.1 ± 5.4	<0.0001	4.0 ± 0.7	0.2064	5.8 ± 0.8	0.0224	6.7 ± 0.8	0.1181	3.1 ± 0.6	0.4680	2.8 ± 0.3	0.0918	2.7 ± 0.3	0.1000
		TBS	15.6 ± 6.0	0.0016	4.0 ± 0.7	0.1000	5.8 ± 0.8	0.0029	6.6 ± 0.7	0.1214	3.2 ± 0.5	0.7750	2.9 ± 0.4	0.5099	2.8 ± 0.3	0.5992
Right Hemisphere	Left hand	Baseline	22.4 ± 3.8		3.7 ± 0.7		5.5 ± 1.0		6.5 ± 0.9		3.1 ± 0.6		2.8 ± 0.3		2.8 ± 0.3	
		5 Hz	20.4 ± 3.5	0.0027	3.9 ± 0.7	0.0056	5.8 ± 0.7	0.0240	7.3 ± 1.1	0.0062	3.3 ± 0.6	0.0094	2.9 ± 0.3	0.3683	2.8 ± 0.3	>0.9999
		10 Hz	20.1 ± 3.1	<0.0001	4.0 ± 0.6	0.0009	5.8 ± 0.7	0.0240	7.1 ± 1.1	0.0094	3.3 ± 0.5	0.0192	2.8 ± 0.3	0.4304	2.8 ± 0.3	0.5958
		TBS	20.4 ± 3.7	0.0009	4.0 ± 0.7	0.0172	5.8 ± 0.6	0.0037	7.1 ± 0.8	0.0020	3.4 ± 0.6	0.0136	2.8 ± 0.2	0.3884	2.8 ± 0.3	0.6477
	Right hand	Baseline	10.6 ± 1.6		3.7 ± 0.6		5.1 ± 0.7		6.0 ± 0.7		2.9 ± 0.6		2.7 ± 0.3		2.7 ± 0.4	
		5 Hz	10.5 ± 1.2	0.9273	4.0 ± 0.7	0.0266	5.4 ± 0.7	0.0172	6.6 ± 0.9	0.0446	3.0 ± 0.4	0.2024	2.7 ± 0.3	0.7562	2.7 ± 0.3	0.4980
		10 Hz	10.4 ± 1.4	0.5958	4.0 ± 0.5	0.0296	5.6 ± 0.6	<0.0001	6.3 ± 0.9	0.0266	3.1 ± 0.5	0.1893	2.7 ± 0.2	>0.9999	2.7 ± 0.4	>0.9999
		TBS	10.5 ± 1.3	0.5706	3.9 ± 0.7	0.0583	5.4 ± 0.6	0.0037	6.4 ± 0.5	0.0136	3.1 ± 0.5	0.0696	2.8 ± 0.3	0.3488	2.7 ± 0.3	0.9854
	Pooled hands	Baseline	16.5 ± 6.6		3.7 ± 0.6		5.3 ± 0.9		6.2 ± 0.8		3.0 ± 0.6		2.8 ± 0.3		2.8 ± 0.4	
		5 Hz	15.5 ± 5.7	0.0079	4.0 ± 0.7	0.0006	5.6 ± 0.7	0.0006	6.9 ± 1.1	0.0003	3.2 ± 0.5	0.0073	2.8 ± 0.3	0.4280	2.8 ± 0.3	0.6753
		10 Hz	15.3 ± 5.5	0.0002	4.0 ± 0.6	<0.0001	5.7 ± 0.6	<0.0001	6.7 ± 1.0	0.0004	3.2 ± 0.5	0.0136	2.7 ± 0.3	0.6463	2.7 ± 0.3	0.1831
		TBS	15.4 ± 5.7	0.0025	4.0 ± 0.7	0.0012	5.6 ± 0.7	<0.0001	6.7 ± 0.8	<0.0001	3.2 ± 0.5	0.0020	2.8 ± 0.3	0.8264	2.8 ± 0.3	0.5900

This table depicts the TCTs of baseline performances and the TCTs measured during stimulation of the supplementary motor area (SMA). The TCTs are sorted by hemisphere, respective protocol, as well as hand (left/right/both pooled). The p-values refer to comparisons of the specific stimulation TCT to the respective baseline evaluation.

Right hemisphere: Faster performances during stimulation were revealed for analyses of the left hand and both hands pooled (p < 0.05; Table 1). Right-handed runs were not significantly different between baseline and stimulation (baseline TCT 10.6 ± 1.6 s; 5 Hz TCT 10.5 ± 1.2 s [p = 0.9273]; 10 Hz TCT 10.4 ± 1.4 s [p = 0.5958]; TBS TCT 10.5 ± 1.3 s [p = 0.5706]). For left-handed performances, lateral stimulation resulted in slower TCTs compared to medial stimulation for both stimulation with 5 Hz (medial group TCT 20.1 ± 3.6 s; lateral group TCT 20.7 ± 3.5 s [p = 0.0064]) and 10 Hz (medial group TCT 19.7 ± 3.0 s; lateral group TCT 20.5 ± 3.3 s [p = 0.0073]).

Regression and visible coordination problems: The regression model revealed that writing was performed with on average 10.4 s (95%-confidence interval [CI] = [−10.7; −10.1]) shorter TCTs for the right hand compared to the left hand (p < 0.0005). All stimulation protocols seemed to significantly shorten the TCTs by 1.4 to 1.1 s compared to the baseline TCT for writing in the model (p < 0.0005 for all protocols; Table 2). No visible coordination problems (VCPs) were detected.

**Table 2:** Test completion time (TCT) differences for stimulation-related parameters in the multi-level regression analyses.

			Jebsen-Taylor Hand Function Test							Nine-hole Peg Test
			Writing	Simulated page turning	Lifting small objects	Simulated feeding	Stacking checkers	Lifting light objects	Lifting heavy objects	
Right hemisphere (compared to left hemisphere)		TCT difference in s [95%-CI]	−0.09 [−0.38; 0.2]	−0.11 [−0.18; −0.03]	−0.05 [−0.15; 0.04]	0.07 [−0.07; 0.21]	0.04 [−0.04; 0.12]	0.04 [−0.04; 0.12]	0 [−0.03; 0.03]	0.12 [−0.08; 0.31]
		p	0.562	0.005	0.278	0.358	0.332	0.01	0.938	0.241
Right hand (compared to left hand)		TCT difference in s [95%-CI]	−10.39 [−10.68; −10.1]	−0.04 [−0.12; 0.03]	−0.23 [−0.32; −0.13]	−0.68 [−0.82; −0.54]	−0.15 [−0.23; −0.07]	−0.08 [−0.12; −0.04]	−0.08 [−0.11; −0.04]	−1.28 [−1.47; −1.08]
		p	<0.0005	0.252	<0.0005	<0.0005	<0.0005	<0.0005	<0.0005	<0.0005
Interaction hemisphere and hand		TCT difference in s [95%-CI]	0.5 [−0.33; 0.49]	0.12 [−0.08; 0.13]	0.14 [−0.24; 0.03]	0.14 [−0.21; 0.19]	0.11 [−0.19; 0.03]	0.06 [−0.06; 0.04]	0.06 [−0.07; 0.02]	0.31 [−0.38; 0.18]
		p	0.699	0.671	0.131	0.932	0.172	0.697	0.36	0.493
Stimulation target (reference first target)	2	TCT difference in s [95%-CI]	0.16 [−0.2; 0.51]	0.06 [−0.03; 0.15]	−0.03 [−0.14; 0.09]	0.01 [−0.16; 0.19]	0.05 [−0.05; 0.15]	0.01 [−0.03; 0.06]	0.03 [−0.01; 0.07]	0.33 [0.08; 0.57]
		p	0.381	0.227	0.632	0.864	0.33	0.615	0.152	0.008
	3	TCT difference in s [95%-CI]	0.03 [−0.32; 0.39]	0.01 [−0.08; 0.1]	−0.09 [−0.21; 0.03]	−0.05 [−0.22; 0.12]	−0.01 [−0.11; 0.08]	0.03 [−0.02; 0.07]	0.01 [−0.03; 0.05]	0.17 [−0.07; 0.41]
		p	0.853	0.812	0.126	0.571	0.805	0.264	0.58	0.167
	4	TCT difference in s [95%-CI]	0.18 [−0.17; 0.54]	−0.01 [−0.1; 0.08]	−0.05 [−0.17; 0.06]	−0.04 [−0.21; 0.13]	0.02 [−0.07; 0.12]	0.03 [−0.02; 0.07]	0.04 [0; 0.08]	0.21 [−0.03; 0.45]
		p	0.312	0.895	0.368	0.639	0.621	0.256	0.083	0.092
	5	TCT difference in s [95%-CI]	0.02 [−0.34; 0.37]	−0.01 [−0.1; 0.08]	0 [−0.12; 0.12]	−0.11 [−0.28; 0.07]	0.01 [−0.08; 0.11]	0.02 [−0.02; 0.07]	0.02 [−0.03; 0.06]	0.23 [−0.01; 0.48]
		p	0.923	0.767	0.988	0.231	0.781	0.355	0.462	0.057
	6	TCT difference in s [95%-CI]	0.06 [−0.3; 0.41]	0.02 [−0.07; 0.11]	−0.05 [−0.17; 0.06]	−0.1 [−0.27; 0.08]	0.03 [−0.07; 0.12]	0.02 [−0.03; 0.06]	0.01 [−0.03; 0.05]	0.05 [−0.19; 0.29]
		p	0.748	0.653	0.372	0.278	0.598	0.527	0.659	0.687
Stimulation protocol (reference baseline)	5 HZ	TCT difference in s [95%-CI]	−1.23 [−1.52; −0.94]	0.23 [0.16; 0.3]	0.27 [0.17; 0.36]	0.48 [0.34; 0.62]	0 [−0.08; 0.08]	−0.06 [−0.1; −0.03]	−0.06 [−0.1; −0.03]	0.79 [0.6; 0.99]
		p	<0.0005	<0.0005	<0.0005	<0.0005	0.922	0.001	<0.0005	<0.0005
	10 Hz	TCT difference in s [95%-CI]	−1.44 [−1.73; −1.15]	0.28 [0.2; 0.35]	0.38 [0.29; 0.48]	0.38 [0.24; 0.52]	0.03 [−0.05; 0.11]	−0.09 [−0.13; −0.05]	−0.07 [−0.11; −0.04]	0.44 [0.25; 0.64]
		p	<0.0005	<0.0005	<0.0005	<0.0005	0.426	<0.0005	<0.0005	<0.0005
	TBS	TCT difference in s [95%-CI]	−1.12 [−1.41; −0.83]	0.24 [0.16; 0.31]	0.38 [0.29; 0.48]	0.35 [0.21; 0.49]	0.09 [0.01; 0.17]	−0.03 [−0.07; 0.01]	−0.05 [−0.08; −0.02]	0.74 [0.54; 0.93]
		p	<0.0005	<0.0005	<0.0005	<0.0005	0.021	0.126	0.003	<0.0005

This table depicts the regression model based on TCTs gained during baseline performance and stimulation of the supplementary motor area (SMA). The calculated influence on TCT of variables such as hemisphere, hand, hemisphere x hand, stimulation target, and stimulation protocol is given in the form of average difference in seconds with the corresponding 95% confidence interval (CI) and p-values.

#### Simulated page turning

Left hemisphere: Opposite effects for simulated page turning were predominantly revealed when compared to writing. For the left hemisphere, we found significant slowing for right-handed performances during TBS (baseline TCT 3.8 ± 0.7 s; TBS TCT 4.1 ± 0.7 s [p = 0.0362]; Table 1).

Right hemisphere: For the right hemisphere, every comparison obtained showed significant slowing of performances (p < 0.05), except for right-handed performances during TBS (baseline TCT 3.7 ± 0.6 s; TBS TCT 3.9 ± 0.7 s [p = 0.0583]; Table 1).

Regression and visible coordination problems: For simulated page turning, the stimulation of the right hemisphere (independent of the executing hand) seemed to result in slightly shorter TCT of on average 0.1 s (95%-CI = [−0.2; −0.0], p = 0.0050) compared to stimulation of the left hemisphere in the model. All stimulation protocols seemed to slow the TCTs by 0.2 to 0.3 s compared to baseline (p < 0.0005 for all protocols; Table 2). In total, four instances of VCPs occurred (Supplementary Videos S1 and S2).

#### Lifting small objects

**Figure 2:**
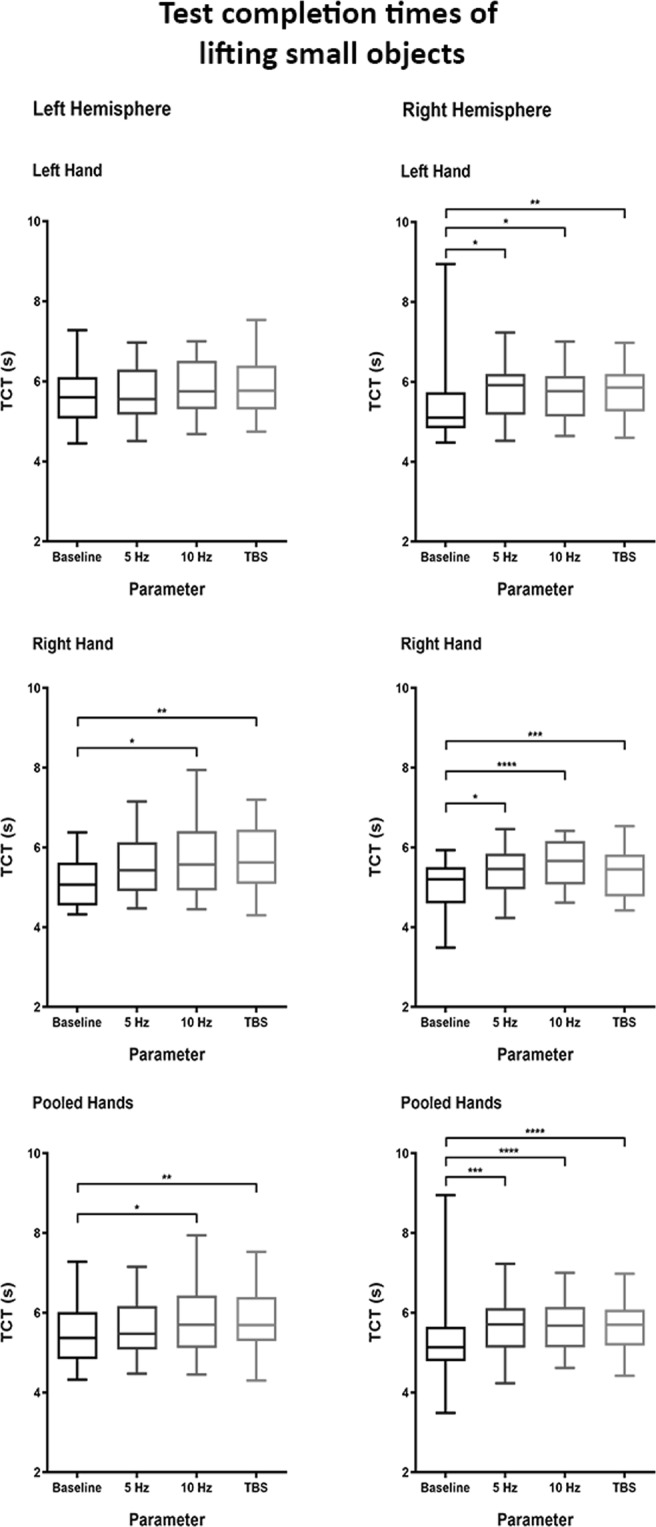
Test completion times (TCTs) of lifting small objects. Boxplots depicting the distribution of TCTs in the task of lifting small objects, separated according to the various analysis pools. For the plots pertaining to left hand and right hand, each single boxplot indicates the distribution of 20 values (one per participant). For the plots pertaining to pooled hands, each single boxplot indicates the distribution of 40 values (the collection of the values from each hand). Whiskers indicate the range of values, boxes depict the two middle quartiles of values. The median is shown by the line inside the box. Testing was done using Wilcoxon rank-sum tests. Statistical significance of differences is indicated by asterisks (cutoffs at p < 0.05, <0.01, <0.001, and < 0.0001 for *, **, *** and ****, respectively).

Left hemisphere: Our analysis discovered significant slowing during stimulation of the left hemisphere with 10 Hz and TBS for right-handed performances (baseline TCT 5.2 ± 0.6 s; 10 Hz TCT 5.7 ± 0.9 s [p = 0.0266]; TBS TCT 5.7 ± 0.9 s [p = 0.0083]) as well as for both hands pooled (baseline TCT 5.4 ± 0.7 s; 10 Hz TCT 5.8 ± 0.8 s [p = 0.0224]; TBS TCT 5.8 ± 0.8 s [p = 0.0029]; Table 1 and Fig. 2).

Right hemisphere: The comparisons focusing on the right hemisphere showed significant slowing in all analyses (p < 0.05; Table 1 and Fig. 2).

Regression and visible coordination problems: For lifting small objects, the regression model showed an in average 0.2 s (95%-CI = [−0.3; −0.1], p < 0.0005) faster performance with the right hand compared to the left hand. All stimulation protocols seemed to slow the TCT by 0.3 to 0.4 s compared to baseline in this test (p < 0.0005 for all protocols; Table 2). Three VCPs were detected (Supplementary Videos S3 and S4).

#### Simulated feeding

Left hemisphere: The statistical analysis showed no significant effect on TCTs through stimulation of the left hemisphere compared to baseline (p > 0.05; Table 1). Within the stimulation paradigm, left-handed performances of 5-Hz stimulation differed between medial and lateral stimulation targets (medial group TCT 6.9 ± 0.8 s; lateral group TCT 7.2 ± 1.1 s [p = 0.0215]). Moreover, for TBS, a rostro-occipital difference could be demonstrated (anterior group TCT 6.7 ± 0.8 s; middle group TCT 6.9 ± 0.8 s; posterior group TCT 7.0 ± 1.1 s [p = 0.0429]).

Right hemisphere: Every comparison during right-hemispheric stimulation demonstrated a significantly slower performance compared to respective baselines (p < 0.05; Table 1).

Regression and visible coordination problems: The regression model also revealed an independent effect for the hand and the stimulation protocols, with 0.7 s (95%-CI = [−0.8; −0.5], p < 0.0005) faster TCT for the right hand than for the left hand, and on average 0.4 to 0.5 s slower TCTs for the stimulation protocols compared to baseline (p < 0.0005 for all protocols; Table 2). Two VCPs occurred in total (Supplementary Videos S5 and S6).

#### Stacking checkers

Left hemisphere: No significant TCT dissociations for left-hemispheric stimulation were revealed (p > 0.05; Table 1).

Right hemisphere: Concerning right-hemispheric stimulation, we found significant slowing of TCTs in all comparisons of left-handed performances (baseline TCT 3.1 ± 0.6 s; 5 Hz TCT 3.3 ± 0.6 s [p = 0.0094]; 10 Hz TCT 3.3 ± 0.5 s [p = 0.0192]; TBS TCT 3.4 ± 0.6 s [p = 0.0136]), no significant slowing for right-handed performances (p > 0.05), and only slowing for the comparisons for pooled hands (baseline TCT 3.0 ± 0.6 s; 5 Hz TCT 3.2 ± 0.5 s [p = 0.0073]; 10 Hz TCT 3.2 ± 0.5 s [p = 0.0136]; TBS TCT 3.2 ± 0.5 s [p = 0.0020]); Table 1).

Regression and visible coordination problems: The TCTs for the right hand seemed to be slightly shorter than for the left hand (0.2 s, 95%-CI = [−0.2; −0.1], p < 0.0005). Only for the TBS protocol a significant difference in the TCTs compared to baseline was observed in the model with on average slightly slower TCT of 0.1 s (95%-CI = [0.0; 0.2], p = 0.0210; Table 2). Six VCPs were identified (Supplementary Videos S7 and S8).

#### Lifting light objects

Left hemisphere: For left hemisphere performances, only stimulation with 5 Hz resulted in a significant effect, both in left-handed performances (baseline TCT 3.0 ± 0.4 s; 5 Hz TCT 2.8 ± 0.3 s [p = 0.0240]; Table 1) and in comparisons for pooled hands (baseline TCT 2.9 ± 0.4 s; 5 Hz TCT 2.8 ± 0.3 s [p = 0.0056]; Table 1).

Right hemisphere: No statistically significant dissociations in TCTs emerged for this subtest (p > 0.05; Table 1).

Regression and visible coordination problems: Lifting light objects was performed slightly slower when the right hemisphere was stimulated (0.04 s, 95%-CI = [−0.0; 0.1], p = 0.0100) and faster when the right hand was used (0.1 s, 95%-CI = [−0.1; −0.0], p < 0.0005). Stimulation with 5 Hz and with 10 Hz seemed to slightly shorten the TCT by 0.1 s, respectively (p = 0.0010 and p < 0.0005; Table 2). During this task, the highest total number of VCPs out of all tests occurred, namely twelve errors (Supplementary Videos S9 and S10).

#### Lifting heavy objects

Left hemisphere: The analyses showed no significant difference between baseline and the varying stimulation conditions in any comparisons (p > 0.05; Table 1).

Right hemisphere: No significant differences between baseline and stimulation were present (p > 0.05; Table 1). Our analysis of stimulation region however showed that lateral TBS was associated with higher TCTs than medial stimulation for left-handed performances (medial group TCT 2.77 ± 0.29 s; lateral group TCT 2.81 ± 0.27 s [p = 0.0362]).

Regression and visible coordination problems: The average TCT seemed to be shorter when the right hand was used as shown in the regression model (0.1 s, 95%-CI = [−0.1; −0.0], p < 0.0005). For all stimulation protocols the model revealed a slightly faster performance than at baseline (0.1 s, p < 0.0005 for 5 Hz and 10 Hz, respectively, and p = 0.0030 for TBS; Table 2). A relatively high number of eight VCPs was identified (Supplementary Videos S11 and S12).

### Nine-hole Peg Test

#### Left hemisphere

**Figure 3:**
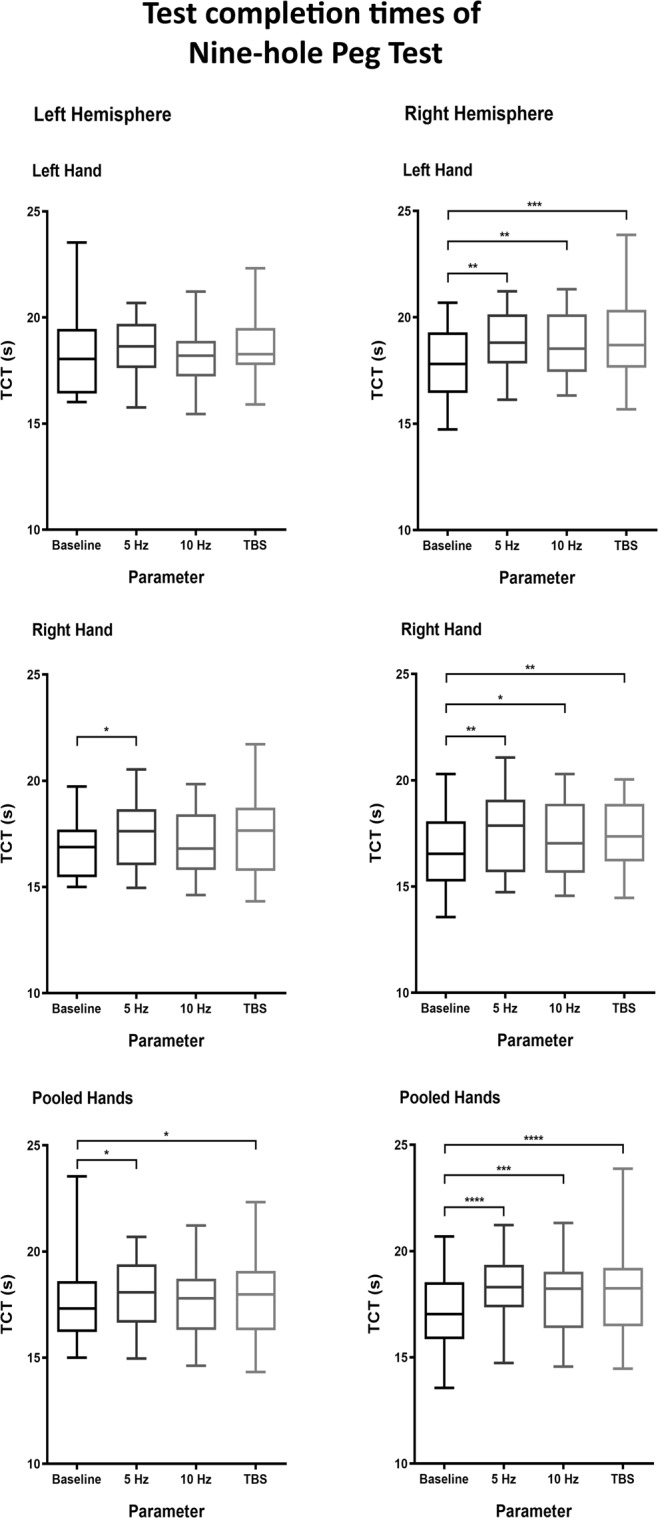
Test completion times (TCTs) for the Nine-hole Peg Test (NHPT). Boxplots depict the distribution of TCTs in the NHPT, separated according to the various analysis pools. For the plots pertaining to left hand and right hand, each single boxplot indicates the distribution of 20 values (one per participant). For the plots pertaining to pooled hands, each single boxplot indicates the distribution of 40 values (the collection of the values from each hand). Whiskers indicate the range of values, boxes depict the two middle quartiles of values. The median is shown by the line inside the box. Testing was done using Wilcoxon rank-sum tests. Statistical significance of differences is indicated by asterisks (cutoffs at p < 0.05, <0.01, <0.001, and < 0.0001 for *, **, *** and ****, respectively).

For the Nine-hole Peg Test (NHPT), we found significantly slower TCTs during left-hemispheric stimulation in comparisons of right-handed performances (baseline TCT 16.8 ± 1.3 s; 5 Hz TCT 17.5 ± 1.6 s [p = 0.0328]) and comparisons for pooled hands (baseline TCT 17.5 ± 1.9 s; 5 Hz TCT 18.0 ± 1.6 s [p = 0.0422]; TBS TCT 18.0 ± 1.8 s [p = 0.0394]; Table 3 and Fig. 3).

**Table 3:** Test completion times (TCTs) for the Nine-hole Peg Test (NHPT).

			Nine-hole Peg Test	
			TCT (s)	p-value to baseline
Left Hemisphere	Left hand	Baseline	18.3 ± 2.1	
		5 Hz	18.6 ± 1.4	0.3884
		10 Hz	18.2 ± 1.3	0.8983
		TBS	18.7 ± 1.6	0.1429
	Right hand	Baseline	16.8 ± 1.3	
		5 Hz	17.5 ± 1.6	0.0328
		10 Hz	17.1 ± 1.6	0.5217
		TBS	17.3 ± 1.8	0.1140
	Pooled hands	Baseline	17.5 ± 1.9	
		5 Hz	18.0 ± 1.6	0.0422
		10 Hz	17.7 ± 1.6	0.7146
		TBS	18.0 ± 1.8	0.0394
Right Hemisphere	Left hand	Baseline	17.7 ± 1.7	
		5 Hz	18.9 ± 1.4	0.0012
		10 Hz	18.6 ± 1.6	0.0049
		TBS	19.0 ± 2.1	0.0006
	Right hand	Baseline	16.6 ± 1.8	
		5 Hz	17.6 ± 1.8	0.0073
		10 Hz	17.2 ± 1.8	0.0484
		TBS	17.4 ± 1.6	0.0014
	Pooled hands	Baseline	17.2 ± 1.8	
		5 Hz	18.2 ± 1.7	<0.0001
		10 Hz	17.9 ± 1.8	0.0005
		TBS	18.2 ± 2.0	<0.0001

This table depicts the TCTs of baseline performances and the TCTs measured during stimulation of the supplementary motor area (SMA). The TCTs are sorted by hemisphere, respective protocol, as well as hand (left/right/both pooled). The p-values refer to comparisons of the specific stimulation TCT to the respective baseline evaluation.

#### Right hemisphere

For stimulation of the right hemisphere, every comparison showed significant slowing during stimulation (p < 0.05; Table 3 and Fig. 3). In left-handed performances, lateral stimulation with 10 Hz corresponded to higher TCTs than medial stimulation (medial group TCT 18.3 ± 1.5 s; lateral group TCT 19.0 ± 1.8 s [p = 0.0136]).

#### Regression and visible coordination problems

A significant effect was demonstrable within the regression model for the right hand with a shorter TCT of in average 1.3 s (95%-CI = [−1.5; −1.1], p < 0.0005). This was the only test where an independent effect for a stimulation target was observed. In this context, stimulation target 2 seemed to have an in average 0.3 s slower TCT compared to stimulation target 1 (95%-CI = [0.1; 0.6], p = 0.0080). All stimulation protocols seemed to slow the performance for this test (by 0.4 to 0.8 s) compared to baseline (p < 0.0005 for all protocols; Table 2). The analysis revealed nine VCPs in total (Supplementary Videos S13 and S14).

### Finger tapping test

Analysis of the standard deviation of inter-tap intervals (SDITs) during the finger tapping test showed significantly more variable inter-tap intervals during stimulation in all comparisons (p < 0.05; Table 4), except for TBS over the right hemisphere (baseline SDIT 67.7 ± 41.3 ms; TBS SDIT 73.8 ± 19.0 ms [p = 0.0826]; Table 4). No VCPs were registered.

**Table 4:** Standard deviation of inter-tap intervals (SDITs) of finger tapping test.

		Finger tapping test	
		SDIT (ms)	p-value to baseline
Left Hemisphere	Baseline	63.1 ± 40.2	
	5 Hz	77.5 ± 29.2	0.0484
	10 Hz	73.3 ± 32.2	0.0266
	TBS	79.6 ± 36.5	0.0441
Right Hemisphere	Baseline	67.7 ± 41.3	
	5 Hz	88.1 ± 35.2	0.0064
	10 Hz	86.5 ± 45.7	0.0400
	TBS	73.8 ± 19.0	0.0826

This table represents an overview regarding the SDITs calculated from the finger tapping test in baseline and under stimulation of the supplementary motor area (SMA). The SDITs are sorted by hemisphere and respective baseline/stimulation condition, with p-values indicating statistical significance.

### Pronator drift test, finger-nose test, and flexion-extension test

No difficulties during performance of the pronator drift test, finger-nose test, or flexion-extension test during baseline assessments or during the stimulation conditions were observed.

### Comparison of stimulation parameters

Comparison of the CIs of the TCT differences for the three stimulation protocols to baseline revealed no clearly superior protocol since CIs were overlapping for all tests (Table 2).

## Discussion

We hypothesized that nTMS to the SMA causes measurable effects on the task performance, which we documented via TCTs, SDITs, and video recordings of VCPs. We were able to demonstrate significant differences of TCTs between baseline and stimulation condition (p < 0.05) in six of seven subtests of the JHFT (except for lifting heavy objects) and in the NHPT, which are both established tests to assess fine motor skills. SDITs were significantly higher under stimulation in the finger tapping test. VCPs were detected most prominently during lifting light objects, lifting heavy objects, and during the NHPT. No effects of stimulation in the pronator drift test, flexion-extension test, or finger-nose test were detected. Our multi-level regression model did not demonstrate one stimulation protocol to be clearly more effective when compared to the other protocols based on the analysis of TCTs. Also, our model did not show clear differences of effect when comparing individual stimulation sites. We were able to determine six instances of lateral targets leading to stronger slowing of TCTs than medial targets. Notably, stimulation of the right hemisphere was able to influence the performance of both executing hands.

To explain the observed results, we would like to point out the concept of the virtual lesion. It assumes that stimulation pulses are able to interfere with physiological computing inside the target structures, thereby eliciting momentary deficits^44^. Analogously, SMA disruption via TMS has been shown in other incarnations, e.g. as degradation of bimanual movement or decline of force control^16,19^. The challenge for the clinical approach with regards to these objective criteria is now to infer a rule for classification of a given point as “SMA-positive”. In this regard, we would argue for a combination of a time-based classification with the more accessible classification via VCPs.

In certain tasks, most notably lifting light objects, lifting heavy objects, and the NHPT, VCPs became apparent (Supplementary Table 1). This again may relate to cognitive control (Supplementary Videos S9–S14). In lifting light objects and lifting heavy objects, we frequently noticed participants using the wrong limb to lift the last can when nTMS was applied (Supplementary Videos S9–S12). All of these occurred seemingly unconscious. The VCPs of the NHPT took a different form. Here, the disruption seems to show itself either as problems in selecting and executing an appropriate movement (Supplementary Videos S13 and S14) or as akinesia, where participants did not initiate any movement for the second part of the task. The former mistakes might resemble the alien-limb syndrome, a condition known to be associated with SMA damage, in which conscious control of a limb is lost^15,45^. The latter could probably reflect acute inability to accommodate to the new part of the task, which is congruent with some studies involving the SMA in attention and performance monitoring^46–48^. Due to this correlation, we consider the occurrence of these VCPs as a positive sign for SMA disruption. This would in turn clearly mark a given target as “SMA-positive”. Due to the ease of detection of these mistakes, we suggest including them in future approaches of nTMS-based SMA mapping.

In our study, the most notable instances of seeming performance facilitation under stimulation occurred during the JHFT subtest of writing (Table 1). Our regression model showed a significant acceleration of TCTs during all three stimulation protocols, which was independent of the stimulated hemisphere (Table 2). However, we are hesitant to interpret this as a verified sign of performance amelioration through nTMS. Looking at studies that examined the practice effect during multiple run-throughs of the JHFT, a quickening in TCTs seems more likely to be due to a practice effect^49^. While we did not implement any specific measurements or corrections for practice effects in this study, their presence remains an important factor. However, studies on practice effects in the JHFT are rare and limited to only one study including 20 women, which also only tested practice over the course of three runs^49^. Meanwhile, our study does contain at least 19 (baseline + 6 targets * 3 protocols) repetitions per hand. In this context, our data might gain new aspects. Since during right-hemispheric stimulation no significant acceleration of TCTs was observed for the right hand, the possibility of right-hemispheric stimulation preventing a learning effect emerges. This would fit the current body of research, which puts emphasis on the role of the SMA in the learning of motor tasks^2,50^. Interference with this function could explain the observed lack of a practice effect. This interpretation does, however, not explain the potentially persisting practice effect for writing with the left hand.

Our data may also be regarded as a qualitative finding regarding lateralization of movement control. Different approaches have led to the model of a rostro-caudal gradient of rising movement lateralization towards occipital direction^1–3^. Our data might affirm this because stimulation of one hemisphere was in some cases able to influence both right-handed as well as left-handed performances (Tables 1 and 3). Furthermore, the absence of a clear interaction between the stimulated hemisphere and hand regarding TCTs can as well be interpreted in this line of thought (Table 2). If there was strong lateralization on the level of the SMA, we should have been able to observe specific effects of hemisphere-hand relations. However, this was not the case, potentially indicating a less strict lateralization.

Furthermore, we would like to point out the difference between the hemispheres. Stimulation of the right hemisphere was more likely to significantly slow down performances than stimulation of the left hemisphere and resulted in stronger slowing in general (Tables 1 and 3). Within the current literature, the body of research investigating interhemispheric differences between the SMA of both hemispheres is small. Some studies found a strong lateralization of inhibitory control functions toward the right hemisphere with a pronounced activation of right SMA in cognitive control tasks^51,52^. While our data fits this model, it is conflicting with other studies. Resection of the SMA in the left or right hemisphere can rather easily be compensated by the respective contralateral SMA^9^. Our data in turn would imply a more severe deficit when resecting SMA of the right hemisphere. A relevant factor in this regard is our focus on right-handed individuals, which would suggest left-hemispheric dominance. Hemispheric dominance has been linked to the apparition of aphasia following SMA resection. This again conflicts with the importance our data places on the right-hemispheric SMA. One might hypothesize that the cognitive control aspect has to be viewed separately from the pure motor aspect. To further shed light on this issue, a study focusing on left-handed individuals might prove to be useful in determining the influence of hemispheric dominance.

Out of the seven significant analyses in which the effect of target location (anterior / middle / posterior targets and medial / lateral targets, respectively) was examined, six showed a stronger effect of lateral stimulation compared to medial stimulation. This could potentially relate to the complex of SMA somatotopy. Many studies point to a somatotopic organization, specifically a rostro-occipital sequence of orofacial, upper extremity, and lower extremity movements^53,54^. The present study was unable to demonstrate this gradient. Instead, we observed a difference between medial and lateral stimulation targets. In preclinical experiments on monkeys, mesial areas have been demonstrated to contain more movement representations than the convexities of the hemispheres^54^. There could be the possibility that by stimulating laterally, mesial parts of the SMA are costimulated. However, the mentioned difference was observed only rarely and without consistent relation to hemisphere, hand, or stimulation parameter. With all this in mind, we are hesitant to assume a stronger stance on the topic of SMA somatotopy within the context of this study. However, this finding may be taken into account when looking for the most effective way to influence SMA activity in general.

No clear difference in effect between the stimulation protocols could be objectively determined (Table 2). Current literature indicates that our protocols should lead to different modulations of activity. TBS is known to lessen activity over time while higher frequencies should rather heighten activation^42^. Considering this, we cannot rule out the possibility of a nocebo effect taking place. However, due to both the frequency of similar VCPs and their correlation with current models of SMA function, we consider this to be unlikely. Taking our findings together, our observations lead us to presume that both inhibiting and activating protocols are able to induce transient and measurable effects on the SMA. One factor possibly elevating TBS over other protocols could be the fact that it has specifically been used for rapid affection of neuronal activity via comparatively high stimulus frequency and has performed slightly better than the other protocols in eliciting VCPs. Due to the very slight differences though, more research regarding 5-Hz and 10-Hz stimulation protocols is still required.

Regarding the study’s limitations, several points have to be raised. First, the lack of a control condition (e.g., targeted stimulation to a region outside of the SMA and primary motor area) has to be considered a relevant shortcoming. Inclusion of such a control condition might have allowed to assign observed effects to the distinct effect of stimulation with more certainty. While we cannot entirely rule out the influence of confounding variables such as the sensation of stimulation, the strong parallels to symptoms connected to SMA dysfunction make an unspecific effect seem unlikely. Both in monkeys and humans, failed coordination of limbs and involuntary movements, similar to the observed VCPs of this study, have arisen out of damage or dysfunction to the SMA^45,55,56^. Furthermore, a recent study applying direct cortical stimulation to premotor areas during awake craniotomy found that the employed stimulation was able to disrupt coordination of hand muscle groups^57^. Thus, real effects of stimulation on SMA-related function seem evident, and stimulation-induced effects have been found consistently using the same electric-field-navigated TMS system regarding other brain functions. For the second limitation, our sample size includes only 20 participants. This limits the generalizability of our findings to some degree. Our statistical analyses, however, imply that the effects we found are robust and more participants would not necessarily add statistical value. Third, our participants were exclusively right-handed according to the Edinburgh Handedness Inventory. We can therefore not expand our findings to the total population and must await more research into the aspects of hemispheric dominance. Fourth, our participants give us data only for the conditions in healthy brains. We can currently make no statements as to the applicability in patients suffering from brain tumors, epilepsy, or taking any kind of neuroactive medication. Fifth, while looking at data from both hemispheres and both hands is necessary in investigating SMA-related phenomena, this also introduces a high amount of complexity to the valid interpretation of data. Nevertheless, we are presently able to demonstrate that there are no difficulties regarding general feasibility of an nTMS-based SMA mapping procedure. While the entirety of the applied tasks would likely prove too overbearing for clinical usage, a reduction to the most promising tests would be very usable.

In conclusion, this study adds to the growing number of investigations of SMA function. Specifically, identification and selective manipulation of SMA via nTMS mapping could enable new approaches in neurophysiological settings to investigate the area’s involvement in its many supposed functions^16,17,52,58^. Moreover, this study contributes evidence to a hemisphere-dependent bilateral motor influence of the SMA by showing stronger disruptions arising from the stimulation of the right hemisphere. Furthermore, we found and statistically confirmed multiple instances of impacted fine motor function during nTMS. These are expressed by higher TCTs and higher SDITs to VCPs. This further builds up the viability of an nTMS-based mapping protocol.

## Materials and Methods

### Ethics

The present study was approved by the local institutional review board (Ethics Committee of Technical University Munich) and was conducted in accordance with the Declaration of Helsinki. Written informed consent was obtained from all subjects. All participants gave explicit informed consent to publication of any video material collected within the context of this study including identifying information/images in an online open-access publication.

### Participants and study design

Twenty healthy volunteers (8 males and 12 females, median age: 22.5 years, age range: 19–30 years) participated in this study. For inclusion criteria, we defined age of at least 18 years, informed consent, and right-handedness according to the Edinburgh Handedness Inventory^59^. Exclusion criteria were pregnancy, contraindications for magnetic resonance imaging (MRI) or TMS (e.g., metallic implants), and any history of neurological or psychiatric diseases. In our analysis, we partly used data previously published within a smaller study in which we focused on ten female volunteers and exclusively on the effects of 10-Hz stimulation regarding the performance during execution of the JHFT^36^.

Each participant first underwent anatomical MRI at 3 Tesla (Achieva; Philips Healthcare, Best, The Netherlands) to acquire a three-dimensional T1-weighted gradient echo sequence (repetition time/echo time: 9/4 ms, 1 mm^3^ isovoxel covering the whole head), used for neuronavigation during later nTMS. Procedures by nTMS were then performed in the context of two separate appointments, which were scheduled at least 14 days apart in each participant. Each appointment was dedicated to motor and SMA mappings of one hemisphere, with the sequence of hemispheres stimulated, single tests, hands (in case of tests for unilateral performance), and order of stimulation of predefined targets being subject to randomization. Apart from these randomizations, the approach of motor and SMA mappings as well as the performance and analyses of tests applied during stimulation were identical during both appointments.

For all nTMS procedures, an electric-field-navigated TMS system was used in order to provide the highest possible accuracy (Nexstim eXimia NBS system, version 4.3; Nexstim Plc., Helsinki, Finland)^60^.

### Motor mapping and determination of targets for SMA mapping

Prior to SMA mappings, motor mapping by nTMS using single-pulse stimulation was performed to delineate the primary motor cortex according to current practice^61^. First, the rMT was determined considering electromyography (EMG) recordings of either the abductor pollicis brevis muscle (APB) or abductor digiti minimi muscle (ADM) using pregelled surface electrodes (Neuroline 720; Ambu, Ballerup, Denmark). The cortical motor hotspot was first identified and then utilized for rMT determination using the built-in procedure considering the maximum likelihood algorithm^62–64^. After the rMT was determined, motor mapping took place considering EMG recordings from electrodes attached to the APB, ADM, flexor carpi radialis muscle, and biceps brachii muscle, with a stimulus intensity of 105% of the individual rMT^61^. The motor mapping of each hemisphere took place directly before SMA mapping, as did the determination of the respective rMT, which was individually assessed in each appointment per participant.

Motor mapping was used to spatially enclose the whole extent of primary motor representations particularly within the superior frontal gyrus (SFG) and middle frontal gyrus (MFG). During analysis of motor mapping data, manual review of mapped points took place, with points marked as motor-positive when the corresponding EMG recording showed plausible latency for upper extremity muscles (15 to 30 ms) and amplitudes of at least 50 µV^61^. Stimulated points not fulfilling these criteria were defined as motor-negative and not considered as part of the primary motor cortex in this study.

For SMA mapping, six stimulation targets per hemisphere were manually placed into the presumed SMA outside of the determined motor cortex delineated by nTMS motor mapping, bordering next to the most anteriorly located motor-positive stimulation spot (Fig. 1)^36^. This was done to ensure that induced motor impairment during SMA mapping could be attributed to SMA stimulation without being confounded by possible stimulation of very anterior parts of primary motor cortex representations^36,65,66^. Prior reports have indicated that the primary motor cortex can extend far anteriorly and beyond the precentral gyrus, with resection of very anterior motor-positive stimulation spots causing postoperative motor deficits related to the primary motor cortex^65^. The number of targets was chosen to allow for complete extension over the anatomical region corresponding to the SMA, while at the same time remaining far enough apart to allow for allocation of effects to each specific target (without presumed stimulation overlap). The targets were generally placed within the posterior SFG, in some cases bordering on the posterior MFG, thus corresponding to the location of pre-SMA and SMA proper as reported in the literature^2^. Inter-target distance was 5 to 10 mm (Fig. 1)^36^. For analysis purposes, the targets were named as follows: posterior targets were targets 1 (medial) and 2 (lateral), middle targets were targets 3 (medial) and 4 (lateral), and anterior targets were targets 5 (medial) and 6 (lateral).

### SMA mapping

#### Test descriptions and baseline assessments

During initial baseline assessments and the SMA mappings, we used the following standardized batteries and tests of movement and coordination:

- JHFT (Sammons Preston, Bolingbrook, Illinois, USA), consisting of seven subtests: writing, simulated page turning, lifting small objects, simulated feeding, stacking checkers, lifting light objects, and lifting heavy objects,
- NHPT (Patterson Medical, Bolingbrook, Illinois, USA),
- Pronator drift test (participants were instructed to lift and hold their arms horizontally in front of them),
- Finger-nose test (participants were instructed to, with their eyes closed, touch the tip of their noses with alternating hands),
- Finger tapping test (participants had to reproduce a simultaneously metronome-generated rhythm of 1 Hz by pressing a key on a keyboard, alternating between the left and right hand), and
- Flexion-extension test (participants had to perform alternating anti-phasic flexing and extending of their arms in a 1-Hz rhythm as given by a metronome).

Baseline assessments (performance without simultaneous stimulation) for these tests were carried out shortly before SMA mappings and subsequent to precise instructions by the examiner and one practice run for each of the above-mentioned tests. For each subtest of the JHFT and for the NHPT, one baseline performance is included in the supplementary videos to this study for the ease of understanding (Supplementary Videos S1–S14). Each test performance was started on command of the examiner. Participants were further told to aim for both a fluent and precise performance, keeping the given rhythm in tests where a metronome was used. All tests were audio- and video-recorded for further detailed analysis after the test procedures.

#### Stimulation of the SMA

**Figure 4:**
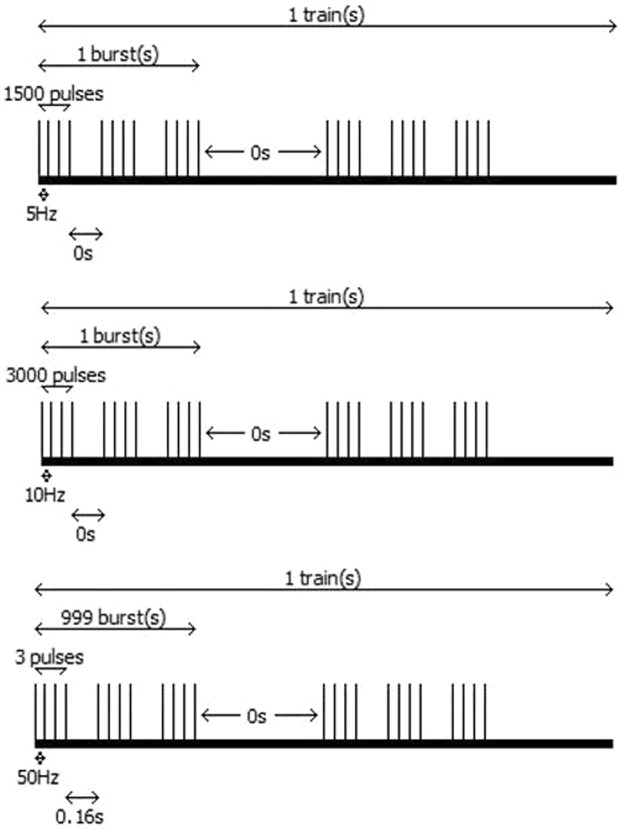
Schematic presentation of stimulation protocols. This figure shows the applied stimulation protocols. From top to bottom, 5-Hz stimulation, 10-Hz stimulation, and theta burst stimulation (TBS) are schematically illustrated with corresponding frequencies and relevant timing details. Stimulation was only applied for the duration of each test performance.

After baseline assessments, SMA mapping was carried out using three different stimulation protocols (Fig. 4):

- 5 Hz (100% rMT, delivered with 1,500 pulses per burst, 1 burst per train, 1 train per sequence),
- 10 Hz (100% rMT, delivered with 3,000 pulses per burst, 1 burst per train, 1 train per sequence), and
- TBS (100% rMT, delivered with 50 Hz, 3 pulses per burst, 160 ms between bursts, 999 bursts per train, 1 train per sequence)^43,67^.

The selected parameters fall under current safety guidelines for stimulation with conventional and patterned TMS outside the motor cortex, where, however, currently no universal limit for safe application has been published^68^. The total duration of each protocol was set so that the stimulation was long enough to cover the full test performance for each test (length >3 min of each protocol). The coil was hand-held during stimulations. Through real-time neuronavigation we ensured optimal conditions for stimulation, keeping the stimulating coil perpendicular to the skull and maintaining a 90° angle of the induced electric field to local gyrus orientation, while at the same time keeping the maximum of the electric field fixed on the respective stimulation target during the task performance^60,69,70^. After each performance, test objects were rearranged into starting constellation, and the coil was moved to the next stimulation target.

After acquisition of one performance for each target, the test was repeated in similar fashion, but executed with the other hand before continuing with the next test. For the entire procedure of SMA mapping including prior motor mapping and baseline acquisition, approximately 270 minutes were needed per participant and appointment, including several breaks to minimize fatigue effects. Total number of stimuli applied was about 35,000 per session (count estimation depending on chosen stimulation protocols and the individual time needed for completion of the tasks).

#### Evaluation of test performances

All test performances under the baseline and stimulation conditions were recorded as video files using the integrated camera of the nTMS system, which allows recording time-locked to the nTMS pulse onset. The camera was placed to allow for full view of the participant performing the tasks, including the test equipment. The following criteria were documented for single tests and considered during later post-hoc evaluation of performances:

- TCTs; time between start command and finishing of a test – JHFT and NHPT,
- SDITs; spread of different inter-tap intervals as gauge for rhythm-keeping ability – finger tapping test, and
- VCPs; qualitative indicators of SMA disruption, such as forgetting the task, inability to move, less fluid movements, significantly worse rhythm keeping – JHFT, NHPT, finger tapping test, flexion-extension test, finger-nose test, and pronator drift test (Supplementary Table 1). VCPs were noted immediately after occurrence by the conducting personnel and with the help of video material.

### Statistics

GraphPad Prism (version 7.0; GraphPad Software Inc., La Jolla, California, USA) was used to calculate descriptive statistics of the cohort and stimulation-related parameters and to generate graphs. Shapiro-Wilk tests confirmed non-normal distribution of TCTs and SDITs.

We compared the rMT between hemispheres by a paired t-test. Wilcoxon rank-sum tests were used to compare the TCTs (JHFT, NHPT) and SDITs (finger tapping test) of the baseline performances to the performances during stimulation. In this context, we compared the average TCTs or SDITs under stimulation (mean of the data gained from all six stimulation targets) to the respective TCTs or SDITs of the baseline condition, which was achieved separately for the mapping of the left and right hemisphere considering the three different stimulation protocols and test conductions with the left and right hand, respectively. Furthermore, we formed additional analyses for pooled data of both hands per stimulated hemisphere and stimulation protocol.

Additional analyses were performed to investigate possible TCT differences between stimulated regions. To this end, we formed three analysis groups based on rostro-occipital orientation (anterior [targets 5 & 6], middle [targets 3 & 4], and posterior [targets 1 & 2], Fig. 1) and two groups based on medio-lateral orientation (medial [targets 1, 3 & 5] and lateral [targets 2, 4 & 6], Fig. 1). We then used Friedman tests to detect possible TCT differences between anterior, middle, and posterior groups. For comparisons of medial to lateral groups, Wilcoxon rank-sum tests were utilized. Within these analyses, stimulation frequency, stimulated hemisphere, and tested hand were always compared correspondingly (e.g., medial left-hemispheric, left-handed 5-Hz stimulation compared to lateral left-hemispheric, left-handed 5-Hz stimulation).

Regarding VCPs (JHFT, NHPT, finger tapping test, flexion-extension test, finger-nose test, and pronator drift test), absolute frequencies were determined by counting such errors, with no statistical method being applied for further evaluation. We did not automatically include instances of dropped test objects, but focused on clear events of movement arrest, limb confusion, or visible decrease in fine motor skills.

For each test a multi-level regression model was generated, with the TCT as the dependent variable and the stimulated hemisphere, hand, stimulation target, and stimulation protocol as the independent variable. An interaction term between hemisphere and hand was added to the model to test for effect modification. To account for dependencies between different test settings for one patient, random effects for patients were added. The regression models were run with the statistical software R (version 3.1.0; https://cran.r-project.org; The R Foundation for Statistical Computing, Vienna, Austria). The corresponding results are given within a 95%-CI.

## Supplementary information

Supplementary information

Supplementary information

Supplementary information

Supplementary information

Supplementary information

Supplementary information

Supplementary information

Supplementary information

Supplementary information

Supplementary information

Supplementary information

Supplementary information

Supplementary information

Supplementary information

Supplementary information

## Data Availability

The datasets generated during and/or analyzed during the current study are available from the corresponding author on reasonable request.
