# Supplementary material for: Navigated transcranial magnetic stimulation of the supplementary motor cortex disrupts fine motor skills in healthy adults
Source: Sci Rep. 2019 Nov 28;9:17744. doi: 10.1038/s41598-019-54302-y (PMC6883055; doi:10.1038/s41598-019-54302-y)
Supplement: Supplementary file 15 — Supplementary information [file 41598_2019_54302_MOESM15_ESM.docx]

**Navigated transcranial magnetic stimulation of the supplementary motor cortex disrupts fine motor skills in healthy adults**

Severin Schramm**^1^**; Lucia Albers, PhD**^1^**; Sebastian Ille, MD**^1,2^**; Axel Schröder**^1^**; Bernhard Meyer, MD**^1^**; Nico Sollmann, MD, PhD**^1,2,3,^***; Sandro M. Krieg, MD, MBA**^1,2,^***

*****: these authors contributed equally

**^1^** Department of Neurosurgery, Klinikum rechts der Isar, Technische Universität München, Munich, Germany;

**^2^** TUM-Neuroimaging Center, Klinikum rechts der Isar, Technische Universität München, Munich, Germany;

**^3^** Department of Diagnostic and Interventional Neuroradiology, Klinikum rechts der Isar, Technische Universität München, Munich, Germany.

**Address for correspondence:**

Sandro M. Krieg, MD, MBA Sandro.Krieg@tum.de

Department of Neurosurgery, Klinikum rechts der Isar,
Technische Universität München, Ismaninger Str. 22, 81675 Munich, Germany
Phone: +49 89 4140 9482, Fax: +49 89 4140 4889

**Supplementary Material**

| Test | Characterization of observed VCPs | 5 Hz | 10 Hz | TBS |
| --- | --- | --- | --- | --- |
| Simulated page turning | Problems in properly gripping pages, Attempted usage of contralateral limb | 2 | 1 | 1 |
| Lifting small objects | Problems in gripping small objects  Dropping of objects outside of target can | - | 2 | 1 |
| Simulated feeding | Dropping of beans outside of target can | 2 | - | - |
| Stacking checkers | Knocking over built towers,  Building of tower outside target ground | 1 | 2 | 3 |
| Lifting light objects | Usage or attempted usage of contralateral limb | 4 | 3 | 5 |
| Lifting heavy objects | Usage or attempted usage of contralateral limb | 1 | 4 | 3 |
| Nine-hole Peg Test | Premature stop of performance after insertion of all pegs,  Inability to use adequate movement for insertion of pegs | 4 | 2 | 3 |

**Supplementary Table 1: Overview over visible coordination problems (VCPs)**

This table shows a characterization of the VCPs as observed in the corresponding tests, as well as their absolute frequencies split by stimulation protocol. Exemplary samples are shown in the Supplementary Videos S1-S14.

**Supplementary Video S1:**

MP4 video material of simulated page turning, showing baseline performance with right hand.

**Supplementary Video S2:**

MP4 video material of simulated page turning during 10 Hz stimulation of left hemisphere, right handed performance. Note the difficulties in executing the task efficiently. The participant herself realizes these difficulties.

**Supplementary Video S3:**

MP4 video material of lifting small objects, showing baseline performance with right hand.

**Supplementary Video S4:**

MP4 video material of lifting small objects during 10 Hz stimulation of left hemisphere, right handed performance. The participant struggles to pick up the second and last object.

**Supplementary Video S5:**

MP4 video material of simulated feeding, showing baseline performance with left hand.

**Supplementary Video S6:**

MP4 video material of simulated feeding, during 5 Hz stimulation of right hemisphere, left handed performance. The participant is unable to coordinate the spoon to the opening of the can and shifts the can, thereby losing the bean.

**Supplementary Video S7:**

MP4 video material of stacking checkers, showing baseline performance with right hand.

**Supplementary Video S8:**

MP4 video material of stacking checkers, during 10 Hz stimulation of right hemisphere, right handed performance. The participant suddenly performs the task incorrectly, stacking the pieces all in one consecutive movement off the board. The participant also realizes her mistake.

**Supplementary Video S9:**

MP4 video material of lifting light objects, showing baseline performance with right hand.

**Supplementary Video S10:**

MP4 video material of lifting light objects, during 5 Hz stimulation of right hemisphere, right handed performance. The participant involuntarily tries using the wrong limb to finish the task.

**Supplementary Video S11:**

MP4 video material of lifting heavy objects, showing baseline performance with left hand.

**Supplementary Video S12:**

MP4 video material of lifting heavy objects, during TBS protocol applied to left hemisphere, left handed performance. The participant involuntarily uses the wrong limb to finish the task and notices his mistake in the end.

**Supplementary Video S13:**

MP4 video material of Nine-hole Peg Test (NHPT), showing baseline performance with left hand.

**Supplementary Video S14:**

MP4 video material of Nine-hole Peg Test (NHPT), during 5 Hz stimulation of the left hemisphere, left handed performance. The participant is visibly unable to continue the task after a certain point, showing problems in executing an appropriate movement.
